# A Review on Recent Advances in Stabilizing Peptides/Proteins upon Fabrication in Hydrogels from Biodegradable Polymers

**DOI:** 10.3390/pharmaceutics10010016

**Published:** 2018-01-18

**Authors:** Faisal Raza, Hajra Zafar, Ying Zhu, Yuan Ren, Aftab -Ullah, Asif Ullah Khan, Xinyi He, Han Han, Md Aquib, Kofi Oti Boakye-Yiadom, Liang Ge

**Affiliations:** 1 Department of Pharmaceutics, China Pharmaceutical University, Nanjing210009, China; faisal@stu.cpu.edu.cn (F.R.); zhuyingsxwd@163.com (Y.Z.); ryuan1993@163.com (Y.R.); 17751032359@163.com (A.-U.); asif.xhan@gmail.com (A.U.K.); hexinyi_cpu2011@163.com (X.H.); hhan_cpu2017@163.com (H.H.); mdaquib0007@yahoo.com (M.A.); otiboakye1000@gmail.com (K.O.B.-Y.); 2 Department of Pharmacy, Quaid-i-Azam University Islamabad, Islamabad45320, Pakistan; hajra.leo1992@gmail.com; 3 State Key Laboratory of Natural Medicines, China Pharmaceutical University, Nanjing 210009, China

**Keywords:** hydrogels, peptides, proteins, crosslinked networks, controlled release, biodegradable polymers

## Abstract

Hydrogels evolved as an outstanding carrier material for local and controlled drug delivery that tend to overcome the shortcomings of old conventional dosage forms for small drugs (NSAIDS) and large peptides and proteins. The aqueous swellable and crosslinked polymeric network structure of hydrogels is composed of various natural, synthetic and semisynthetic biodegradable polymers. Hydrogels have remarkable properties of functionality, reversibility, sterilizability, and biocompatibility. All these dynamic properties of hydrogels have increased the interest in their use as a carrier for peptides and proteins to be released slowly in a sustained manner. Peptide and proteins are remarkable therapeutic agents in today’s world that allow the treatment of severe, chronic and life-threatening diseases, such as diabetes, rheumatoid arthritis, hepatitis. Despite few limitations, hydrogels provide fine tuning of proteins and peptides delivery with enormous impact in clinical medicine. Novels drug delivery systems composed of smart peptides and molecules have the ability to drive self-assembly and form hydrogels at physiological pH. These hydrogels are significantly important for biological and medical fields. The primary objective of this article is to review current issues concerned with the therapeutic peptides and proteins and impact of remarkable properties of hydrogels on these therapeutic agents. Different routes for pharmaceutical peptides and proteins and superiority over other drugs candidates are presented. Recent advances based on various approaches like self-assembly of peptides and small molecules to form novel hydrogels are also discussed. The article will also review the literature concerning the classification of hydrogels on a different basis, polymers used, “release mechanisms” their physical and chemical characteristics and diverse applications.

## 1. Introduction

Targeted drug delivery to specific body parts has become one of the important ventures of the today’s world as conventional dosage forms are generally associated with difficulties in approaching the target site at the specified dose after or during a proper time period. As a result, the search for novel drug delivery systems and a new mechanism of action has become very active. Novel drug delivery systems comprise of lipidic, proteic and polymeric technologies that provide a controlled and sustained drug delivery with better pharmacokinetics, stability towards the harsh external environment and avoid rapid clearance of drugs. Many of these advances have reached the market therefore as new drug carriers [1]. Many drawbacks are associated with conventional drug delivery system, e.g., poor patient compliance, which leads to missing the frequent doses of drugs with a shorter half-life. A typical sawtooth pattern of plasma concentration-time profile is observed which makes attainment of steady-state concentration very difficult. There is also unavoidable fluctuations in the drug concentration that may cause under or overdose activity of drugs as the steady state concentration value fall or rise beyond the therapeutic range. Recent research in advanced pharmaceutical preparations thus aims to provide stable and cost-effective drug delivery systems. The main focus is on hydrogels to reduce not only the shortcomings of old conventional dosage forms but also those of novel drug delivery systems to provide a more convenient, compatible and stable drug delivery system for small drug molecules like NSAIDs (Non-steroidal anti-inflammatory drugs) or large molecules as proteins and peptides [2,3]. Hydrogels evolved as an outstanding carrier material for local and controlled drug delivery [4]. Hydrogels have been defined in many different ways by researchers over the past years. One of them, the most common defines hydrogel as a polymeric material that possesses the capability of swelling and retains a certain high amount of water within its structure and does not dissolve in water medium itself [5,6,7].

Initially, poly (2-hydroxyethyl methacrylate) (poly HEMA) was mentioned as a hard, brittle and glassy polymer and it certainly it was not considered of much importance. The intrinsic ability of hydrogels to absorb water is due to several functional groups (such as –NH_2_, COOH, –OH, –CONH_2_, –CONH–, and –SO_3_H) which are hydrophilic and attached to the polymeric chain. Hydrogels are resistant to dissolution and this property arises from cross-links between polymeric chains [8]. Hydrogels have remarkable properties of functionality, reversibility, sterilizability, and biocompatibility which meet fulfill material and biological requirements to treat targeted tissues and organs or replace it or interact with the biological systems [9,10,11]. The important characteristics of these hydrogels are its ability to swell when interacting with water [12].

Hydrogels can be classified as natural, synthetic or semisynthetic, according to the nature of crosslinking polymers. Hydrogels constitute chemical or physical crosslinking of polymers. The hydrogel matrix allows physical incorporation of proteins and its mechanism of release is usually controlled diffusion, swelling, erosion/degradation, or a combination of these. Hydrogels provide fine-tuning of the protein and peptide drugs release by redefining their cross-linking through changes in the polymer structure, its concentration, molecular weight, or chemistry. Other methods to tailor drug release from hydrogel matrix are also known that involve reversible protein−polymer interaction or protein encapsulation in a secondary delivery system as micro/nanoparticles dispersed in the hydrogel matrix [13,14]. Hydrogels have enormous uses in clinical medicine and experimental settings for a wide range of applications, including tissue engineering and regenerative medicine [15], diagnostics [16], cellular immobilization [17], separation of biomolecules or cells [18], and barrier materials to regulate biological adhesions [19].

Although hydrogels have many advantageous properties, several limitations are also associated with these materials. They have a poor tensile strength which could limit their use in drug loading applications and can result in the premature dissolution or flow away of the hydrogel from targeted tissues and organs. This drawback is of much importance in many typical topical and subcutaneous drug delivery technologies. The amount and homogeneity of hydrophobic drugs are also minimized in hydrogels. Another disadvantage is the presence of high quantity of water and large pore sizes in most hydrogels which often result in relatively rapid drug release, in few hours. The application of hydrogel can also be problematic sometimes; though some hydrogels are ultra-deformable to be injected, many are not, thus need surgical insertion. Each of these issues critically limits the practical use of hydrogel drug delivery systems in the clinical applications [20]. Hydrogel innovation is also linked to problems such as solubility, high crystallinity, non-biodegradability, unfavorable mechanical and thermal properties, unreacted monomers and the use of toxic crosslinkers. Therefore, the improvement of these properties can be possible with the use of a combination of natural and synthetic polymers with enhanced properties [21].

Self-assembly is also used in the generation of hydrogels with significantly improved properties. Molecular self-assembly comprises many non-covalent interactions with which basic molecular building blocks organize spontaneously and reversibly into a novel, supramolecular and functional nano-scale manner. Peptides and proteins are useful building blocks for supramolecular hydrogels and can combine structural and functional activity [22].

Self-assembling peptides serves as attractive candidate for the development of hydrogels with well-controlled biological, mechanical and material properties. Peptide-based hydrogels offer numerous advantages such as their easy synthesis, characterization and decoration, biodegradability and most importantly their very high biocompatibility [23]. Current findings shows that relatively short peptides (di-, tri- and tetra-peptides) can freely self-assemble into ordered nanostructures including hydrogels, that have made this area of research very dynamic and exciting [24]. The spontaneous self-assembly of a dipeptide, Leucine-α,βdehydrophenylalanine, containing a non-protein amino acid, α,β-dehydrophenylalanine (ΔPhe) at its C-terminal, into a highly stable hydrogel under physiological conditions. ΔPhe is an analogue of phenylalanine, with a double bond between Cα and Cβ atoms, whose incorporation in peptide sequences introduces conformational restriction in the peptide backbone and provides increased resistance to enzymatic degradation [25,26]. The hydrogel designed by LeuΔPhe was transparent, self-supportive, fractaline in nature, of high mechanical strength, non-toxic, injectable, proteolytically stable and responsive to external stimuli, such as ionic strength, pH and temperature. Fibrilar network of the dipeptide gel could encapsulate and release numerous hydrophobic and hydrophilic drug molecules in a controlled manner. The gel restored its original strength after disturbance of its structure, showing its thixotropic behaviour. Administration of the antineoplastic drug, mitoxantrone, entrapped in LeuΔPhe hydrogel in tumor bearing mice, significantly controlled growth of tumors and improved the antitumor activity of the drug. These distinctive characteristics of this low molecular weight dipeptide hydrogel make it an exciting candidate for further improvement as a drug delivery platform. Several other supramolecular self-assemblies of small molecules proteins are also reported to have supportive interaction. Several other self-assembling peptide have been reported, including those based on d- and l-amino acids, which have several advantages [27,28]. Hydrophobic drugs, such as NSAIDs, can be delivered in a prolong manner with these systems if they participate to the self-assembly process and are thus bond non-covalently to the supra molecular structure [29]. Examples of enzyme in-structure self-assembly include phosphatase and thermolysin-based self-assembly of small molecules. These enzymes catalyse hydrolysis to trigger self-assembly. Similarly for small molecule bounded proteins the supramolecular material is attached with photoreactive motif that drive the self-assembly. This in turn transform into transparent hydrogel that can retain several proteins, such as tubulin, actins and several others [22].

Peptides and proteins are known for years as complex structures. Naturally, there are twenty different amino acids join together peptide bonds and build chains known as peptides and proteins. Several processes as fermentation, purification and recombination technology led to the production of potential proteinaceous drugs in an economical way which isused in wide range of diseases. They can be administered through various routes like oral, transdermal, nasal, pulmonary, ocular, buccal, and rectal. By making the availability of these pharmaceutical proteins and peptides possible these drugs can prove to safe and effective therapeutics. Due to its large applications in pharmaceutical fields, they will probably take the important place of organic-based pharmaceuticals. In recent years, therapeutic peptides and proteins have reached a successful level. Several diseases can be treated with this type of therapeutics include auto-immune diseases, cancer, mental disorder, hypertension, and certain cardiovascular and metabolic diseases. Recombinant technology has made possible the production of potential proteins and peptide drug it possible in a cost-effective way. This allows the treatment of severe, chronic and life-threatening diseases, such as diabetes, rheumatoid arthritis, hepatitis in an easy manner. Currently, over 160 protein drugs are available on the world market, and several hundred are on way in clinical trials. The total market for protein and peptide drug market has crossed 30 billion and expected to increase at 10% per year at least. The therapeutical peptide and proteins are have gained a place of important therapeutic agents rapidly. The peptides and protein-based drugs will be produced on a large scale by biotechnology processes and available on market for therapeutic use soon. The benefits of having favorable time to market and high level of success in clinical applications in comparison with conventional pharmaceuticals, therapeutical peptides and proteins will play the main role in the treatment of various ailments [30,31].

## 2. Pharmaceutical Peptides and Proteins

Novel sustained release formulations of peptides and proteins provide better opportunities for the cure and prevention of disease. These formulations can affect the chemical integrity and three-dimensional structure of proteins during manufacture. Various polymers are used for sustained release of these proteins and peptides the most important of which includepoly (lactide-co-glycolide) (PLG) [32]. The peptide and protein-based drugs are effective in treating various kinds of life-threatening diseases. Improvements in treatment and cure are made clear by changing the properties of peptides and proteins. To this end various approaches can be used one of which can be structural modification. Alteration in the structural moieties of these drugs changes solubility, lipophilicity, stability, crystallinity, targetability, taste and enzymatic susceptibility [33]. Proteins and peptides are chemically and enzymatically unstable. Enzymatic degradation affects both the fraction absorbed and also the half-lives of peptides in the body [34].

Peptides/Proteins have a short half-life and immunogenicity risks and many strategies have been developed. The PEGylation process of conjugating proteins or peptides with polyethylene glycol (PEG) can improve the pharmacokinetics of the drug and increase the plasma half-life of the protein. This increases the hydrophilicity of the molecule so that it is not recognized by macrophages and it prevents opsonization. PEGylation also reduces the immunogenicity that the protein might possess [35].

## 3. Hydrogels

Biodegradable hydrogel provides an alternative mechanism of drug release from the polymer matrix [36]. The released of the entrapped drug can be controlled by altering the degradation rate of the hydrogel. By making the hydrogel degradable only in the existence of certain enzyme, the biodegradable hydrogel can release drug at a specific site in the body. The products produced from the degradation of some polymers, such as Poly (L-lactic acid) (PLLA) and Poly (lactic-co-glycolic acid) (PLGA), may lower the pH of the adjacent environment. Such degradation products may cause inflammation at the injection site. pH/temperature-sensitive block copolymer hydrogels show numerous advantages over thermosensitive block copolymer hydrogels, such as the absence of blockage during injection, which allows facile injection into deep sites in the body, prevention of the local low pH environment caused by degradation, which protects proteins/cells from damage, and the ease of handling and storage [37].

### 3.1. Smart Hydrogels

Smart hydrogels are attractive new hydrogels that are composed of materials able to undergo transitional changes in response to environmental stimuli [38]. These transitional changes include swelling, shrinkage, degradation, or a sol to gel phase transition when exposed to external physical or chemical stimuli, such as changes in pH, temperature, solvent, pressure, ionic strength, light, and concentration of specific biomolecules [38]. Environmental triggers provide specific functions, such as controlled drug release, protein separation, and muscle activity, or in situ gelling systems.

### 3.2. pH Sensitive Hydrogels

The pH range occurring at physiological, pathological, or subcellular sites such as the stomach, intestine, endosome/lysosome, and tumor sites is used for the formation of these hydrogels. Polymers used in this process are those with weak polyelectrolyte (polyacid, polybase) or polyampholyte sequences [39]. pH sensitive hydrogels with ionic groups accept or donate protons with respect to changes in environmental pH change. The degree of ionization (pKa or pKb) changes in response to pH change. pH change leads to sudden volume transition that creates large osmotic swelling force. For example, the polymer polydiethylaminoethyl methacrylate and its co-polymers ionize in response to pH change. Two types of pH-responsive hydrogels are known: anionic and cationic hydrogels. Anionic hydrogels have attached groups of carboxylic or sulfonic acid. Here deprotonation followed by ionization occurs when the surrounding pH is above the pKa which induce swelling of the hydrogel [40,41,42]. On the other hand, cationic hydrogels have group such as amine groups, where pH below the pKb is required for ionization and swelling [43,44].

### 3.3. Temperature Sensitive Hydrogels

**Figure 1:**
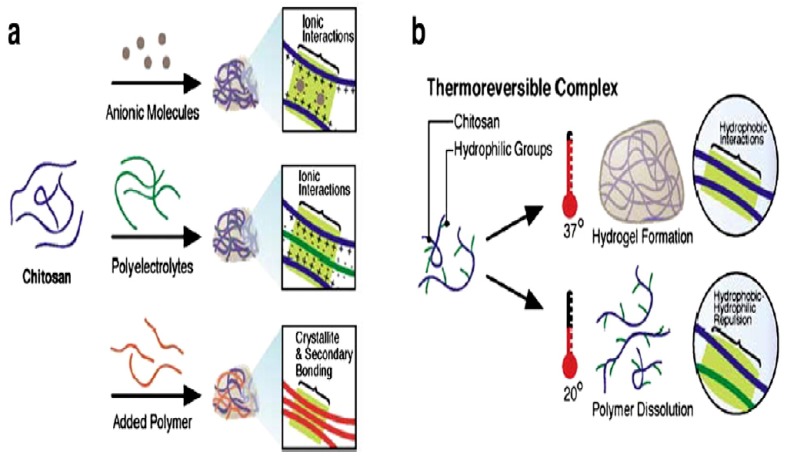
Chitosan-basedthermosensitive hydrogel (**a**) networks of chitosan formed with ionic molecules, polyelectrolyte polymer, and neutral polymers; (**b**) thermo-reversible networks of chitosan graft copolymer resulting semi-solid gel at body temperature and liquid below room temperature. Reprinted from [46]. Copyright (2018), with permission from Elsevier.

Temperature sensitive hydrogels are unique to swell and shrink as temperature changes in the environment. Their swelling and deswelling behavior mostly is dependent on surrounding temperature. Chitosan-based thermosensitive hydrogels are best in situ polymeric solution for injectable purposes Figure 1. Upon heating their hydrophobic regions are readily formed and connected to for yield a stable structure. This hydrophobic effect is observed to be the main driving force for thermosensitive gelation. These thermosensitive hydrogels possess good fluidity, thermosensitivity, and biodegradability. Temperature sensitive hydrogels can be classified as positive or negative temperature responsive systems [45].

#### 3.3.1. Positive Temperature Hydrogels

Positive temperature hydrogels are hydrogels with specific upper critical solution temperature (UCST) [47]. Temperature above upper critical solution temperature is required for swelling of hydrogels. Whereas at a temperature below the UCST, dehydration occurs and the hydrogels shrink and release solvents or fluids from the matrix. Such types of hydrogels are retrogressive at negative temperatures. Positive temperature hydrogels shrink at low temperatures because of a complex structure formation by the hydrogen bonding. A number of hydrogels formed by Interpenetrating polymer networks (IPN) show positive thermosensitivity i.e., they shrink at low temperatures and swell at high temperatures. Poly (acrylic acid) and polyacrylamide (PAAm) or P (AAm-co-BMA) performs as positively thermosensitive hydrogels.

#### 3.3.2. Negative Temperature Hydrogel

This type of hydrogel is known as low critical solution temperature (LCST). Shrinkage occurs as temperature increases above the LCST and shows a swelling below LCST. The LCST is the most critical parameter for these hydrogels and can be altered in different ways including mixing of a small amount of ionic copolymer in the gels or by changing the composition of the solvent. Their hydrophobic constituent shifts them to lower temperatures [48]. The ratio of hydrophilic and hydrophobic constituents controls the LCST. Hydrogen bonds are formed at a temperature below LCST when water interacts with the hydrophilic part and this improves swelling and dissolution behavior. As the temperature rises above LCST, the hydrophobic interaction occurs and the hydrogen bonds become weaker that leads to shrinking of hydrogel [49]. Example of negative hydrogel includes Polyvinylpyrrolidon/poly(*N*-isopropylacrylamide) (PVP/PNIPAM).

### 3.4. Glucose-Sensitive Hydrogels

**Figure 2:**
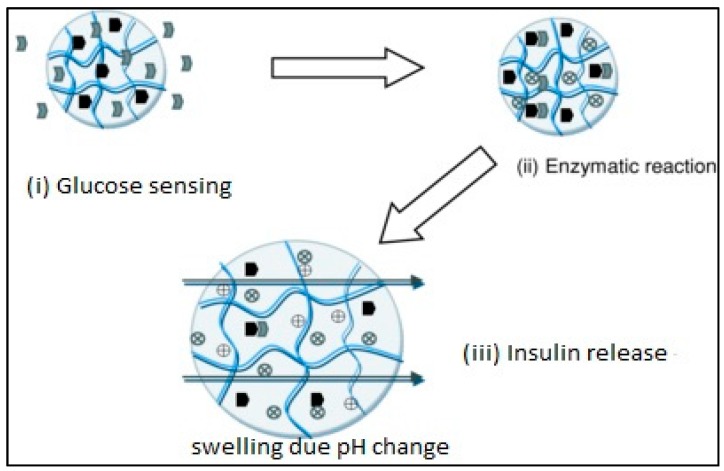
Glucose-sensitive hydrogel mechanism. Reprinted from [52]. Copyright (2018), with permission from Elsevier.

These hydrogels respond to change in glucose concentration. For example, insulin hydrogel used for the treatment of diabetes responds to the glucose to initiate the release of insulin. It also contains glucose sensor suitable for insulin delivery. Glucose-sensitive hydrogels are attractive insulin carriers with novel technology [50,51]. The swelling of hydrogel triggers the release of insulin when the local pH of the system reduces when glucose is converted to gluconic acid by glucose oxidase enzymes in the presence of oxygen. Glucose oxidase has been covalently attached to hydrogel network for controlling the release of insulin. These hydrogels are composed of materials that are “bio-smart”, in which engineered molecular recognition site is linked with actuation, consisting of HEMA and PMA. The local pH of the system is reduced when glucose is converted to gluconic acid by glucose oxidase in the presence of oxygen, which increases the swelling of cationic hydrogels and releases insulin as shown in Figure 2.

### 3.5. Protein-Based Hydrogels

**Figure 3:**
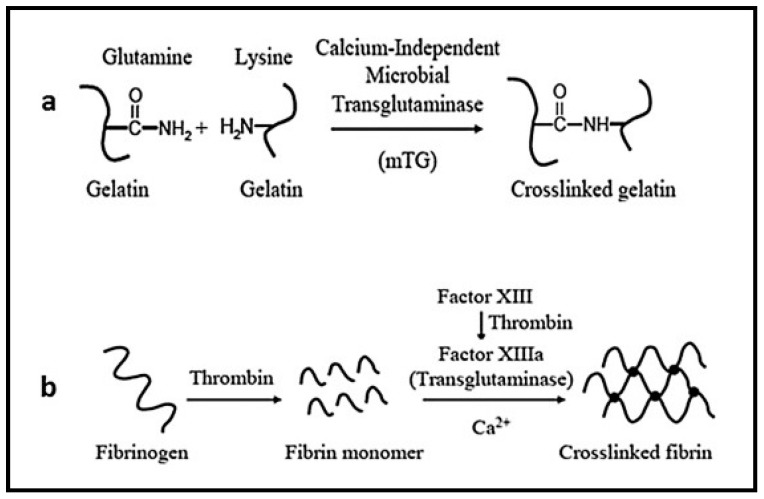
Protein-based hydrogels. Reprinted from [54]. Copyright (2018), with permission from Elsevier.

Protein-based hydrogels are composed of special sequences, stereochemistry, and molecular weights. These are developed by using recombinant DNA technology for drug delivery and tissue engineering applications. One of best approaches for protein-based hydrogels includes coiled-coil method. In these hydrogels, the hydrophobic amino acid groups of the coiled-coil proteins are used in physical crosslinking of gels as shown in Figure 3. Physically crosslinked protein-based hydrogels are composed of tri-block copolymers with coiled-coil domains at the end and water-soluble polypeptide domains in the center [51]. By modifying the amino acid sequences in coiled-coil domain stimuli sensitivity to temperature and pH can be achieved Moreover, 3D structure of the hydrogel is also possible when water-soluble linear synthetic polymer coiled-coil proteins are used as crosslinkers [53].

### 3.6. Antigen-Responsive Hydrogels

An antigen sensitive hydrogel is prepared by grafting antigen and the respective antibody to the polymer network, where they bind to initiate crosslinks into the network [55]. When there is no free antigen the chain antigen binds to the antibody and cause shrinkage of the hydrogel. They can be used as a useful carrier for biomolecules and deliver proteins at the target site through antigen sensing technique [52].

### 3.7. Monomers Used for Fabrication of Hydrogels

**Figure 4:**
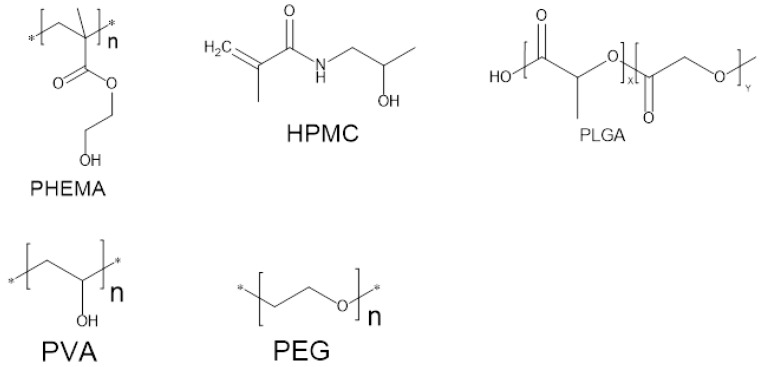
Common monomers frequently used in hydrogels.

A range of monomers is used for the fabrication of hydrogels which include several novel materials with tailored characteristics suitable for particular applications. The first synthesis of hydrogel was carried out by Wichterle and Lin using PHEMA (Poly hydroxyethyl methacrylate) as the monomer. Monomers of thehydrogel are selected based on their properties, ease of delivery or encapsulation as well as cost and availability considering their applications. Commonly used monomers include polyvinyl alcohol (PVA), polyethylene glycol (PEG) and methacrylate as shown in Figure 4. Another most common monomers used for drug delivery of protein from hydrogels is biodegradable PLGA (polymer of lactic and glycolic acid). However, PLGA is composed of hydrophobic materials and it denatures the proteins as well as cause inflammation due to degradation. By using hydrophilic polymers these problems can be overcome. For example acrylic acid, polyethylene glycol and methacrylic acid are all materials with hydrophilic nature, are used for therapeutic purposes. Researchers are now trying to synthesize materials suitable for specific applications. PNIPAAm (poly *N*-isopropyl acrylamide), PVA (polyvinyl alcohol) are all prepared by new techniques, for newer applications [56]. Table 1 Provide a list of some common monomers used for biomaterial synthesis [21].

### 3.8. Loading of Drugs in Hydrogel

Drugs can be loaded into hydrogel matrices in two ways [57]:

*(a)*  Post-loading
*(b)*  In situ loading

First one is the post loading. In the post-loading method the polymer and drug are mixed first then initiators and crosslinker are added to polymerize the gel with the drug inside the matrix [58]. On the other hand in the in situ loading, a preformed hydrogel is placed in a drug solution and allowed to swell till equilibrium. In both, the method the hydrogel is dried after drug loading. Drug loading within hydrogel is dependent on several factors including polymer and solvent interaction, cross-linking density of the polymeric network, the presence of a solvent etc. All these factors affect the ration of swelling to a greater extent [59]. The drug release from hydrogel matrix will be determined by mechanisms like diffusion, hydrogel swelling, reversible drug-polymer interactions or degradation of sensitive covalent bonds [57,60,61,62,63,64,65].

### 3.9. Release Mechanism of Drug from Hydrogel Matrices

According to various modelistic studies on the possible release mechanisms of an active agent from a hydrogel matrix, and considering the rate-limiting step of the release phenomena, the drug release mechanisms from hydrogels can be categorizedas:

1. Diffusion-controlled
2. Swelling-controlled and
3. Chemically-controlled.

Diffusion-controlled is described by Fick’s law (with constant or variable diffusion coefficient) as the most common and dominant mechanism for drug release [66]. Drug release by diffusion depends upon mesh sizes of the matrix material of hydrogel [67], which, in turn, is controlled by several factors such as the degree of crosslinking, chemical structure of the monomers and intensity of the external stimuli [66,67,68]. Usually, mesh sizes from 5 to 100 nm (in their swollen state) have been reported for biomedical hydrogels [68,69], which are much larger than most of the small-size drugs. These mesh sizes retard the release of several drugs while large macromolecules like oligonucleotides, peptides, and proteins show sustained release pattern. These mesh sizes can be custom design to allow the sustained release of macromolecules [70]. The second one is the swelling-controlled mechanism, in which diffusion of a drug is much faster. Swelling is considered to control the drug release behavior [71,72]. The last one is the chemically-controlled release where chemical reactions occur within the gel matrix. These reactions include breakage of polymer chain through hydrolytic or enzymatic degradation or reversible/irreversible reactions in the polymer network. These reactions tend to release the drug from the matrix. Several other mechanismsare also known for the controlled release of the drug. These include a surface or bulk erosion of hydrogels or the binding equilibrium among the drug binding moieties loaded within the hydrogels [21,66,73].

### 3.10. Proteins/Peptides Stability in Hydrogels

A successful drug delivery system depends upon the ability to release active proteins, as well as providing their sustained release. During the evaluation hydrogel-based system the assessment of protein stability must be implemented to verify pharmacological drug activity and the lack of immune reactions. The stability of the protein must be maintained during the preparation, storage, and release of the hydrogel. The major issue in hydrogel formulation includes maintenance of protein’s structure due to incomplete release due to aggregation, chemical binding between protein and polymer, oxidation, deamidation, etc. [74]. Studies reported that oxidation affects the stability of bovine serum albumin (BSA) and interleukin loaded in hydrogels. The extent of oxidation can be reduced by the addition of an antioxidant. Post loading of proteins in gels can be used as a method to prevent interactions and to avoid their unwanted chemical modification [75]. Protein loading into polymer carriers can lead to loss of native 3D structure and denature proteins. Denatured proteins usually tend to aggregate. Their altered structure can induce immune reactions such as antibodies production that neutralize the activity of the therapeutic protein [76]. Protein denaturation and aggregation have been found in protein/PLGA preparations [77]. As hydrogel have high water portion which provides better compatibility with proteins as compared to hydrophobic part like PLGA. Also, there is limited mobility of proteins in hydrogel matrices that further contributes to the stability of entrapped therapeutic proteins entrapped proteins. Many studies also reported that the structure and bioactivity are retained after it is released from the hydrogel. In general, hydrogels protect proteins from denaturation/aggregation more than other types of matrices. Peptides and proteins are freely metabolized in the gastrointestinal tract (GIT), and the hydrophilic nature of most natural peptides limits movement through the epithelial barrier as shown in [78]. Advances in the oral delivery of proteins and peptides have been made by the use of absorption enhancers, enzyme inhibitors and direct structural modification of the therapeutic. Mucoadhesive polymers, nanoemulsionsand NPs have been used to increase the stability of peptides as well to increase their absorption. Peptide and protein therapeutics, with their high target specificity and wide applicability, have the potential to develop medical therapy [78].

A wide diversity of hydrogels has been designed to date, changing from gels based on natural proteins such as collagen and gelatin, to hybrid hydrogelscontaining PEG as the polymeric scaffold. Amodel candidate in this respect would be a hydrogel composed of natural peptides or proteins, combined with a synthetic scaffold. In this way, proteins like elastin and collagen will afford biocompatibility and will introduce biological functionality. The mechanical and structural properties of the hydrogelcan be altered by incorporation of synthetic polymers. This givesrise to gels with proper strength and shear thinning behavior [79]. The great variety of amino acid sequences delivers the possibility to fine-tune supramolecular interactions to form hydrogels of numerous properties. These hydrogels own a great potential as active biologics carriers due to their inherent biocompatibility and tunable biodegradability [80]. Drug-loadedsupramolecular hydrogels increase the potential for sustained drug delivery. The supramolecular scaffolds control the release of the therapeutic agent at the target site and improve the bioavailability to several extents. The supramolecular structure of gel also provides stability to the attached therapeutic agent that retains at the target site for a longer period of time.

### 3.11. Recent Advancement in Hydrogel

#### 3.11.1. Supramolecular Hydrogelators and Hydrogels

Supramolecular hydrogels based on peptides are developing medicine, as they are bringing novelty in areas spanning from new therapeutic examples used to direct cell fate [81,82], to enhanced immune response to vaccines and to new tools to help surgical procedures [83]. The development of supramolecular hydrogels in the past two decades not only has underscored the above consequence but also has delivered a fundamentally new method for chemists to control the properties of soft materials via the molecular engineering of a varied set of substrates for an extensive kind of applications [84]. The most important applications of the hydrogels are to mimic the Extra cellular matrix (ECM) for cell culture (or tissue engineering), and the necessary primary test for a hydrogelator is its cell compatibility [85]. Now we discuss several hydrogelators that have been evaluated for the applications related to cells, [86] such as hydrogelators for cell culture [24,87], cell-compatible hydrogelators [88,89], cytotoxic hydrogelators [90], and hydrogels for cell adhesion.

**Figure 5:**
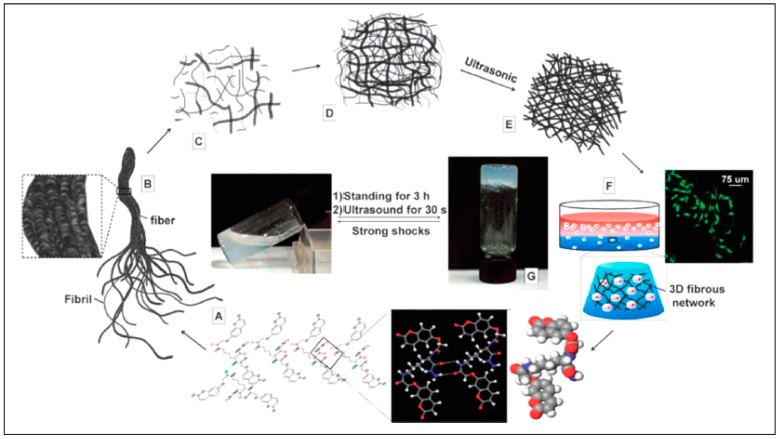
Formation mechanism of hydrogel: (**A**) hydrogen-bond-driven self-assembly; (**B**) self assembled fibrils; (**C**) fibrils with a hydrogelator concentration lower than the minimum gelation concentration (MGC); (**D**) entangled fibrils with a hydrogelator concentration higher than the MGC; (**E**) well-organized 3D hierarchical nanoarchitectures with ultrasound treatment; (**F**) cells seeded in hydrogels; (**G**) optical image of the hydrogel (the transition from solution to hydrogel was reversible). Reprinted from [99]. Copyright (2018), with permission from Royal Society of Chemistry.

Chemo/biosensors for visual exposure are a class of progressively smart tools for the investigation of many targets (e.g., biological markers, enzymes, ions, gases, etc.) [91]. They are really useful for rapid and high-throughput diagnostics or detection in circumstances where low cost, speed, and ease are required. “Stimulus-responsive” or “smart” supramolecular hydrogels, thus, attract consideration as a platform for chemosensors because they have the following properties/advantages: (i) A variety of biological, chemical, or physical Triggers (e.g., temperature, pH, ionic strength, electric field, enzyme, etc.) Instruct the formation of supramolecular hydrogels which report the occurrence of the targets [92]. For example, hydrogel formation triggered by enzymes can Function as an indicator of certain enzymes [93]; (ii) Supramolecular hydrogels are able to incorporate/immobilize a variety of colorimetric reagents, such as visible dyes, both covalently and noncovalently [94]. A variety of diverse yet selective molecular interactions can lead to a color change of the hydrogel, for example, stimulus-induced release or absorbance of dye molecules and color changes of the hydrogels started by target binding; (iii) Supramolecular hydrogels-based chemosensors can work in aqueous conditions, which is of extreme significance because most biological substances (e.g., enzymes, biomarkers, etc.) remain active only in physiological conditions (i.e., in aqueous solution) [95]. Supramolecular hydrogels show solid like, yet soft, properties and comprise three-dimensional networks as shown in Figure 5, formed by hydrogelators, to not only absorb water, but also immobilize other components, such as small molecules, enzymes, and ions, particularly when the hydrogels help as chemo/biosensors. Before discussing supramolecular-hydrogel-based chemo/biosensors, we first highlight some recent works on hydrogels used as effective absorbents of dyes, metal ions, and other molecules [96,97]. Due to the use of an extensive range of dyes in numerous industries (e.g., paper, plastics, textiles, and cosmetics), it is essential to eliminate the dyes from industrial discharge to avoid pollution. Among all kinds of approaches, absorption is more preferred due to its low cost, high efficiency, and easy handling. Supramolecular hydrogels which contain both hydrophilic and hydrophobic groups can absorb a variety of dyes [98] and may have advantage in the recycle and adsorption rate.

As we move towards more sophisticated hydrogel architectures, the improvement in stimuli-responsive systems has the potential to move from large, user-generated stimuli, to smaller, locally generated biological stimuli. The capability to engineer these hydrogel systems to listen and respond to cell-based stimuli can cover the way to the creation of independent biomaterials to be used in tissue engineering, drug delivery, sensing, and cancer therapeutics, among other fields. With smart engineering and design, one can imagine the design of materials that cannot only review the real-time response of the natural ECM, but also move towards multi component systems [100], that offer non-natural or enhanced function [101], controlling the growth, differentiation, and migration of cells towards the construction of regenerated tissues. For example, the design of bioresponsive hydrogels with complex logic gate functions has already been proved [102], and such logic gate designs allow complex responses from complex inputs. The toolbox of chemistries to use in the design of cell responsive systems can be measured small, but more importantly, such chemistries are often poorly studied in the circumstance of complex biological systems. For example, supramolecular biomaterials grasp great capacity to restore and mimic the complex and dynamic extracellular environment [103].

#### 3.11.2. Antibacterial Hydrogelators/Hydrogels

Infectious disease remains the main risk to public health, and there is an urgent need for novel antimicrobial agents with activities against multi-drug-resistant bacteria. The discovery of antimicrobial peptides has stimulated the use of self-assembly of peptide amphiphiles to improve antibacterial hydrogels. Mainly, the innovative work by Schneider et al. on antibacterial hydrogels has delivered useful insights into the development of hydrogelators for antibacterial applications [104]. Schneider and Pochan et al. reported a series of β-sheet peptide-based hydrogels [105], among which the surface is inherently antibacterial and exhibits broad-spectrum activity Against both Gram-negative (*Klebsiella pneumonia* and *E. coli*) and Gram-positive (*Staphylococcus epidermidis*, *Staphylococcus aureus*, and *Streptococcus pyogenes*) bacteria without incorporating Exogenous antimicrobial agents. Using the LIVE/DEAD assays by laser scanning confocal microscopy (LSCM), they found that the surface of the hydrogel of 2 wt. % 251 displays broad-spectrum antibacterial activity when incubated with bacterial solutions ranging in concentration from 2 × 103 to 2 × 109 colony-forming units (CFUs)/dm^2^.

As adjuvants are crucial components of vaccines, harmless and more potent adjuvants are gaining increasing importance and attention. As injectable biomaterials for drug delivery and tissue engineering, supramolecular hydrogels made of peptides or peptide derivatives are outstanding candidates as adjuvants because of their little cost of production, ease of being produced in huge quantities, and comparatively high activity and stability. These advantages of peptides or peptide derivatives promise their applications in cancer immunotherapies and vaccination against infectious diseases, mainly for increasing the potency of vaccines or for delivery of vaccines. Since the building blocks of supramolecular hydrogels are molecules, the successful development of supramolecular hydrogelators or hydrogels as molecular biomaterials demands bioengineers or medical doctors to have a deep understanding of molecular interactions, and chemists to acquire knowledge of molecular and cell biology. For example, the end of the synthesis of molecules and the characterization of molecular structures or supramolecular structures becomes the starting point of the research for chemists, not the ending point. If the goal of the research on the supramolecular hydrogelators is to improve molecular biomaterials, it would be helpful to have a chemist who has the information of molecular biology and cell biology and clinical medicine, is capable to communicate with the language of biochemistry, and possesses the skills of bioinformatics.

It is our belief that the innovative study of supramolecular hydrogelators and hydrogels not only will bring innovative molecular biomaterials but also may lead to new frontiers of science [106]. The amazing flexibility of supramolecular hydrogelators and hydrogels has offered scientists a fruitful playground to reinvest in the context of molecular biomaterials. By shifting the research focus from molecules to processes [107], from thermodynamics to kinetics [108], and from molecules to cells, the research on supramolecular hydrogels and hydrogelators will lead to the integration of molecular science and bioinformatics and contribute to the use of molecules for better human life.

### 3.12. Hydrogels for Pharmaceutical Applications

**Figure 6:**
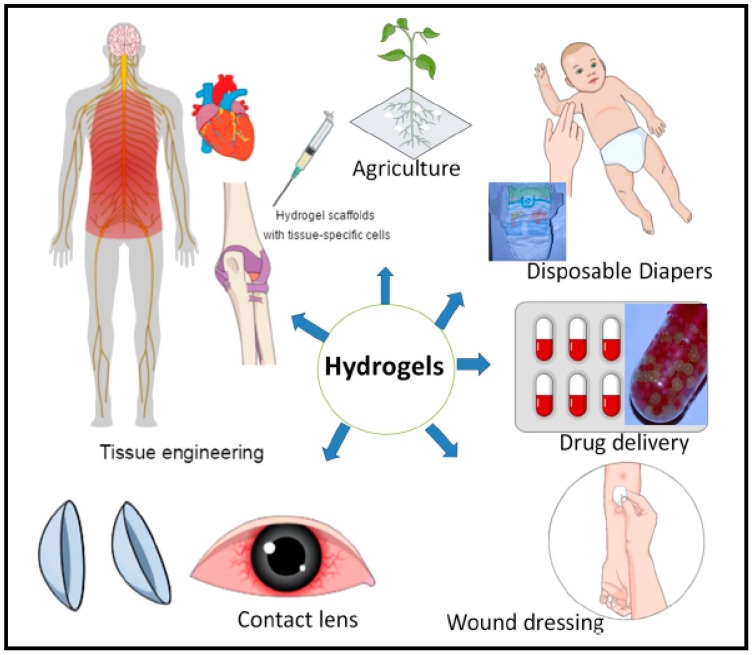
Hydrogels and their significance in their various fields of applications. Reprinted from [117]. Copyright (2018), with permission from Elsevier.

Hydrogels are widely used as biomaterials in several applications due to their structural resemblance to the body tissues Figure 6. For example, drug delivery systems based on hydrogel is of great importance as these are tailored to provide a modified release to the target site, prolong circulation time, and reduce toxicity and side effects. Hydrogels not only serve as an excellent carrier for small molecules but also for delicate bioactive large molecules such as proteins. Due to its high water content, it retains the activity of proteins and protects from denaturation thus making it ideal encapsulation and release material for proteins. Hydrogels serve as ideal drug delivery systems with desirable therapeutic features [109]. They have excellent physicochemical and biological properties with a wide diversity of polymeric materials which lead to excellent candidates for delivery systems of pharmaceutical agents [110,111,112]. Pharmaceutical hydrogels have various categories based on different criteria mainly including, route of administration [113,114], type of material therapeutic agent to be delivered [21,57,113], and release profile [115,116].

Following are some main applications of pharmaceutical hydrogels:

(1) Special hydrogels are composed of superabsorbent materials and they are used as hygienic materials for various purposes such as in disposable diapers and lady napkins and absorb the secreted fluids [118].
(2) Superabsorbent hydrogels are mainly used as targeted drug delivery system and Nano/controlled drug delivery systems.
(3) Polysaccharides-based hydrogels for bioactive coatings (e.g., catheter, stent), replacement of nucleus pulpous and cellular scaffold (artificial organs)
(4) Microporous hydrogels have the capacity for control release of therapeutic agents and for blood purification [119]. They are also used as regenerative medicine for cell delivery and dressing material or wounds [120,121].
(5) Acidic cellulose–chitin hybrid gel that is a novel electrode which is used for electric double layer capacitor.
(6) Hydrogels that are prepared from silk fibers prepared excellent interstitial fluid support capacity and they are employed for articular cartilage repair [122].
(7) Elastin-like protein hydrogels are found to improve neuriteout-growth in neuronal cultures. These hydrogels contain engineered polymers that produce elastin-like proteins recombinant protein containing amino acids.
(8) Elastin-based polymeric hydrogels are mainly utilized for advanced engineering of elastic tissues, such as skin, lung, and vasculature. They are composed of expandable polymeric fibers which facilitate the blood vessels to stretch and relax billion times or more during their lifetime period [123].
(9) Poly (hydroxyethyl methacrylate)-based hydrogels are used to ensures good wound-healing therefore mostly applied in wound dressings, specifically burn dressings applications. It is used for contact lenses, drug delivery and tissue engineering purposes for bone marrow and spinal cord cell regeneration. It also promotes cell adhesion in artificial skin and cartilage manufacturing method [124].
(10) PVA-based hydrogels are used widely in biomedical applications including drug delivery, artificial tears, contact lenses, artificial cell encapsulation and as nerve cuffs [125]. PVA hydrogel also found application in injectable implants, soft tissue fillers, cartilage reconstructive, aesthetic surgery, artificial organs, drug delivery systems and wound dressings. It provides ahumid environment that is useful for wound healing [126].
(11) Poly (ethylene glycol)-based have high biocompatibility, lack of toxic effects on the surrounding tissues and high solubility in water which serve as good candidates for drug delivery systems [124]. They are employed for cell delivery to improve tissue regeneration.
(12) Thermosensitive tri-block poly (ethylene glycol)-poly (ε-caprolactone)-poly (ethylene glycol)-based hydrogels which are formed in situ, can be easily utilized in various biomedical fields such as cell encapsulation, controlled drug delivery, and tissue repair. Poly (imide) (PI) hydrogels and PVA hydrogels are used in plastic and reconstructive surgeries [124].
(13) Polyacrylate (PA)-based polymeric hydrogels play important role in advanced aesthetic corrections, soft tissue fillers and enhancement materials [124].
(14) Poly (urethane) hydrogels serve as drug matrices, artificial kidney membranes, and catheter coating materials [127].

## 4. Conclusions and Future Perspectives

Significant progress has been made in the field of hydrogels as a functional biomaterial. Hydrogels form a promising material for controlled release of pharmaceutical proteins and peptides due to their capacity to incorporate therapeutical agents in the hydrophilic polymeric network. The soft nature, porous structure, and large water content make hydrogels suitable carriers to incorporate a lot of drugs and to provide sustained release for a specified period of time. Their characteristics enable them to be employed as essential tools in almost all fields such as biomedical, agricultural, industrial and environmental areas. These hydrogels are of high interest for the encapsulation and entrapment of bioactive substances. Many cross-linking methods have been devised for hydrogel synthesis. Protein loaded hydrogels are prepared by a wide number of methods with the aim to improve therapeutic efficacy and patient compliance. Most of the preparation methods applied for hydrogels preserver the native structure of proteins and peptides, thus ensuring their stability for long period of time. Stimuli-responsive and smart hydrogels form an attractive approach for non-invasive treatment. Hydrogels provide fine tuning of proteins and peptides delivery with significant results in clinical medicine. The unique features of smart and stimuli-responsive hydrogels can be utilized for the effective pharmacological impact of active therapeutic agents. The soluble polymers make these hydrogel biodegradable and biocompatible. A versatile combination of polymers makes these hydrogels responsive to different stimuli like temperature, pH, and various chemical stimuli. These stimuli-responsive hydrogels are reported to have diverse applications for chronic diseases. In short area of hydrogels is making efforts to bring protein delivery to the clinical application. Moreover, the characteristic properties of hydrogels can be controllable with significant impact on the stability of therapeutic agents.

We further expect an increased knowledge of the composition of hydrogel material will allow controlling the release of more sensitive drugs. The hydrogel can be fabricated from nano-sized particles termed as nanohydrogel, which is expected to provide improved stability to biopharmaceuticals such as peptides and proteins. Nano hydrogels can help to retain the three-dimensional structure of these agents and direct them specifically to the target site. Nano-sized hydrogel formulation can also be expected to minimize enzymatic degradation of peptides and proteins by protecting these agents in the polymeric network. Hydrogel formulation with tunable properties will provide patient-specific treatment that would be highly promising for chronic diseases. There is a need to produce hydrogels with enhanced durability, improved mechanical properties, and significant biocompatibility. Even though biocompatibility issues are resolved, there is still a need for the synthesis and evaluation of biodegradable and aqueous soluble polymers with the aim to improve the efficacy and safety of drug delivery hydrogels. The biosafety and cytotoxicity are other important features that must be evaluated by using specified tests to render these materials safe for drug delivery purposes. Nano hydrogels with smart polymers are considered to fulfill all these properties in near future. The concept ofnanogels with conjugated biomolecules, such as proteins, peptides, and antibodies, can be utilized to target cancerous cells and induce apoptosis thus improving normal cellular functions. Similarly, smart co-polymeric hydrogels can also be considered for the disease modification in diabetes, cardiovascular problems, and immune complications. Scientists are also working on another class of hydrogels with remotely controlled properties. These remote-controlled hydrogel nanocomposites are thought to be highly efficient for clinical applications. Further studies are required in this area to make the remotely controlled hydrogel nanocomposite responsive to external biochemical, physical and chemical stimuli. Protein engineering and chemical modification can also be useful for the modification of characteristics properties of protein loaded hydrogels.

Commercialization of drug delivery systems based on hydrogels and their nanocomposites is required for the efficient utilization in clinical areas and better Pharmaco-economics. This is a further demand for the involvement of industries and healthcare systems. The preparation and evaluation techniques for hydrogels face certain challenges, such as high cost, low durability, and complicated procedures. This extensive research is required is to reliable techniques and overcome the issues. All the variables in the process cycle must be critically evaluated and controlled for optimized results. Composite hydrogels systems can be prepared with less complexity to allow for the commercialization of the product. More attention is needed to meet the specific requirements of advanced drug delivery systems. Several challenges are still a part of advance hydrogel formulations, which have to be overcome for diverse clinical applications in coming years. Focusing on clinical requirements and reducing the complexity of hydrogels are considered to be the main goals form coming decades.

## Figures and Tables

**Table 1:** Monomer used in the synthesis of hydrogel for Pharmaceutical Formulations.

Monomer Abbreviations	Monomers
HPMC	Hydroxypropyl Methylcellulose
HEEMA	Hydroxyethoxyethylmethacrylate
HDEEMA	Hydroxydiethoxyethyl methacrylate
MA	Methacrylate
MEEMA	Methoxyethoxyethyl methacrylate
PLGA	Polymer of lactic and glycolic acid
PHEMA	Poly hydroxyethyl methacrylate
PVA	Polyvinyl alcohol
PEG	polyethylene glycol
PNIPAAm	poly *N*-isopropyl acrylamide
AA	Acrylic acid
MAA	Methyl Acrylic acid
HPMA	*N*-(2-hydroxypropyl)metharcrylamide
PVA	Polyvinyl alcohol
PEGA	PEG-acrylate
PEGMA	PEG methacrylate
PEGDA	PEG diacrylate
PEGDMA	PEG dimethylcrylate
